# Secondary intention healing with biodegradable temporizing matrix (BTM) in a comorbid patient: A case report

**DOI:** 10.1016/j.jpra.2025.11.020

**Published:** 2025-11-23

**Authors:** Ho Yin Kam, Yuet Ching Wong, Stephen Goldie

**Affiliations:** a Department of Plastic and Reconstructive Surgery, Royal Hobart Hospital, Hobart, Tasmania, 7000, Australia; b School of Medicine, University of Tasmania, Hobart, Tasmania, 7000, Australia; c Department of Surgery, Monash University, Melbourne, Victoria, 3168, Australia

**Keywords:** Skin neoplasm, Biodegradable temporizing matrix, BTM, Wound healing

## Abstract

Biodegradable temporizing matrix (BTM) is a synthetic dermal substitute designed for complex wound reconstruction, typically in a two-stage process. We present a case involving BTM-assisted healing by secondary intention of an infected scalp wound in a medically complex patient following failed split-thickness skin grafting. Owing to substantial granulation and neodermis formation, the planned second-stage grafting was ultimately deemed unnecessary. This case highlights BTM’s potential as a definitive reconstructive option in high-risk patients with impaired healing capacity and supports its role in managing complex wounds, particularly when conventional reconstructive strategies are contraindicated or have failed.

## Introduction

Cutaneous squamous cell carcinoma (cSCC) is the second most common cancer in Australia after basal cell carcinoma, accounting for about one-third of non-melanoma skin cancers.^1^ It typically arises on sun-exposed areas, especially the head and neck. Surgical excision is the primary treatment and is curative with adequate margins.^1^ Secondary non-oncological goals of treatment include maintaining function and cosmesis, and often necessitates the use of complex reconstructive techniques, particularly in the case of larger, more aggressive and deeply invasive lesions, such as skin graft or flaps.

The biodegradable temporizing matrix (BTM, Novosorb, PolyNovo Biomaterials Pty Ltd., Port Melbourne, VIC, Australia) is a fully synthetic dermal substitute designed for the reconstruction of complex wounds.^2^^,^ ^3^ It is typically used in a two-stage wound reconstruction process, serves as a transitional phase to optimize the wound bed before grafting.^4^ We present a case report and serial clinical images demonstrating BTM-assisted healing solely via secondary intention of a large, infected scalp vertex wound in a patient with significant comorbidities and previous skin graft failure.

## Case report

**Figure 1:**
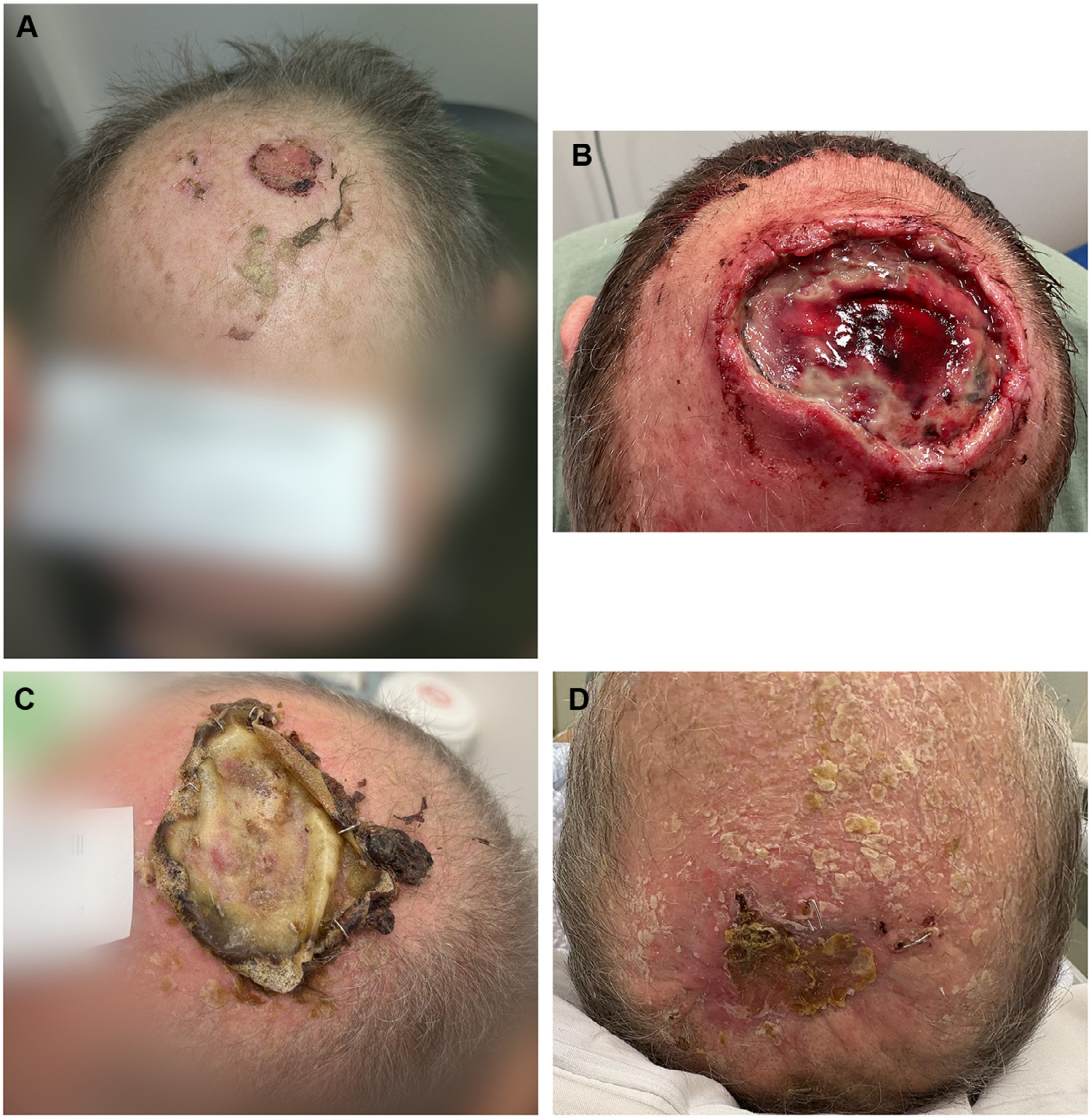
A: Scalp SCC at initial referral. B: 2-week post excision and STSG, with signs of wound infection and complete graft loss. C: 9-week post BTM application, superficial non-degradable layer in situ, no signs of infection. D: 12-week post BTM application & patient self-delamination, healthy pink colour with small central eschar demonstrating good healing via secondary intention.

A 60-year-old male presented with a 25 × 24 mm biopsy proven ulcerated, moderately differentiated squamous cell carcinoma lesion of the scalp vertex (Figure 1A), subsequently underwent an excision of the lesion with split thickness skin grafting (STSG) at the Launceston General Hospital (Tasmania, Australia) in January 2025. He is a co-morbid patient with significant medical history including atrial fibrillation (AF) managed with apixaban, long-standing poorly controlled insulin-dependent type 2 diabetes mellitus (T2DM), ischemic heart disease (IHD), and peripheral vascular disease. He is also a renal transplant recipient since 2015 on long-term regular immunosuppressants (including tacrolimus, mycophenolate and prednisolone). Wound review at 2 weeks excision and STSG revealed surgical site infection and near-complete graft loss (Figure 1B). The wound was debrided in clinic, and the patient was discharged with oral antibiotics and a plan for ongoing silver foam dressing changes coordinated through community nursing services. At 3-week post-op, no improvement was observed and he was therefore re-admitted for a debridement and washout of the wound with application of BTM. The BTM was applied to the entirety of the wound and was inset by staples, followed by Acticoat and Hypafix dressings. Additionally, he was treated with intravenous antibiotics and was discharged Day 2 post surgery.

Due to complex social circumstances, the patient relocated to the Hobart region following the application of BTM and subsequently was followed up by the plastic surgery team at the Royal Hobart Hospital (RHH). Early follow-ups noted episodes of exudation, erythema, and malodor suggestive of infection, however, BTM continued to integrate without signs of wound breakdown.

Delamination of BTM with skin grafting was initially planned at 6-week post-BTM application. During a clinic review at 5-week post BTM application, the patient developed sudden chest pain, prompting urgent admission at the RHH. The admission was complicated by his poorly controlled T2DM and an acute kidney injury, which resulted in a postponement of the initial plan. Once medically stabilized, the patient was followed up by the RHH plastic surgery team in April 2025 (9-week post BTM), which showed continued integration of BTM with overlying non-degradable membrane with no signs of infection, requiring delamination (Figure 1C). At the planned second-stage surgery, the superficial BTM membrane had peeled off spontaneously prior to admission and the wound had nearly completely granulated, with only a small central area of eschar and some peripheral staples in situ (Figure 1D). Given the favorable healing, the second stage was deemed unnecessary. Staples and eschar were removed and wound healing outcome was satisfactory.

Histopathological examination demonstrated a 25 × 24 mm ulcerated, well-differentiated squamous cell carcinoma with a depth of invasion of 8.5 mm into the subcutaneous tissue. All peripheral margins were clear by >10 mm, and the deep margin measured 2.7 mm. No perineural or lymphovascular invasion was identified. According to current guidelines, a microscopic margin of ≥1 mm is considered curative for cSCC.^5^ In this case, the margins were clear and deemed curative. Although adjuvant radiotherapy was considered due to the depth of invasion (>6 mm),^5^ it was ultimately not pursued because of the patient’s complex social circumstances, postoperative wound infection and breakdown, and ultimately loss of the optimal therapeutic window. The patient was therefore managed with clinical surveillance with no signs of recurrence at 5-month post excision.

## Discussion

BTM is a fully synthetic dermal substitute designed for the reconstruction of complex wounds. It comprises a 2 mm layer of biodegradable polyurethane open-cell foam, overlaid with a non-biodegradable sealing membrane.^2^^,^^3^ BTM is typically used in a two-stage wound reconstruction process.^4^^,^^6^ In the first stage, it serves as a temporary wound cover, with fenestrations to prevent fluid buildup and an open-cell matrix that supports cellular and vascular infiltration, leading to neodermis formation. As granulation tissue develops, the matrix biodegrades, leaving a surface suitable for grafting—often with a split-thickness skin graft in the second stage.^4^^,^^6^

This case illustrates a departure from the standard two-stage approach to the clinical use of BTM. The literature does report a few cases with relatively good outcomes of wound healing by secondary intention over BTM over small areas in healthy individuals.^7^^,^^8^ However, an extensive literature search revealed only one case of secondary healing over BTM in a co-morbid patient.^9^ This prompted the decision to document this case to report on the potential benefits of BTM in patients with multiple comorbidities that impair wound healing, particularly those requiring skin graft reconstruction following extensive skin cancer excision.

There is extensive documentation on BTM’s resistance to infection in the literature. Lo et al. demonstrated a high rates of BTM integration, often despite findings of infection,^6^ and similar results are also shown by Greenwood et al. and Solanki et al. demonstrating robust evidence of BTM’s resistance to infection.^4^^,^^10^ This is certainly what we have observed in our case. Despite the patient’s significant comorbidities—including chronic immunosuppression, poorly controlled insulin-dependent T2DM, and clinical features suggestive of wound infection—BTM demonstrated sustained structural integrity and successful integration. Notably, there was no evidence of wound breakdown throughout the treatment course. The matrix supported progressive neodermis formation and granulation, ultimately facilitating wound closure without the need for the planned second-stage skin grafting.

In retrospect, BTM represented the most viable reconstructive option for this patient, and hence it was utilized for this specific case. Given the prior graft failure—likely influenced by infection and significant comorbidities—alternative strategies such as local flaps or repeat grafting carried a high risk of failure. Although the second-stage surgery was ultimately omitted, BTM facilitated satisfactory wound healing, with reasonable cosmetic outcome and patient satisfaction.

## Conclusion

This is a case of successful wound healing by secondary intention using BTM alone, following a failed skin graft, achieved in a patient with multiple comorbidities and impaired healing capacity—representing a favorable clinical outcome. BTM offered effective definitive wound coverage with notable resistance to infection, despite the patient’s limited physiological reserve. This case adds to existing literature suggesting that in comorbid patients requiring graft-based reconstruction, BTM could potentially be considered as the definitive management in less aesthetically sensitive areas to promote wound healing and patient outcomes.

## Patient consent

Patient has given informed written consent to the publication of images and/or data.

## Funding

None.

## Declaration of competing interest

None declared.
